# The Oryx Antelope (*Oryx gazella*): An Unexpected Host for Porcine Circovirus-2 (PCV-2)

**DOI:** 10.3390/pathogens10111402

**Published:** 2021-10-29

**Authors:** Umberto Molini, Lauren Michelle Coetzee, Maria Yvonne Hemberger, Siegfried Khaiseb, Giovanni Cattoli, William G. Dundon, Giovanni Franzo

**Affiliations:** 1 School of Veterinary Medicine, Faculty of Health Sciences and Veterinary Medicine, Neudamm Campus, University of Namibia, Windhoek 13301, Namibia; umolini@unam.na (U.M.); mhemberger@unam.na (M.Y.H.); 2 Central Veterinary Laboratory (CVL), 24 Goethe Street, Windhoek 18137, Namibia; laurenctz13@gmail.com (L.M.C.); Khaisebs@gmail.com (S.K.); 3 Animal Production and Health Laboratory, Animal Production and Health Section, Joint FAO/IAEA Division, Department of Nuclear Sciences and Applications, International Atomic Energy Agency, P.O. Box 100, 1400 Vienna, Austria; g.cattoli@iaea.org (G.C.); W.Dundon@iaea.org (W.G.D.); 4 Department of Animal Medicine, Production and Health, University of Padova, Viale dell’Università, 35020 Legnaro, Italy

**Keywords:** PCV-2c, Namibia, oryxes, epidemiology, molecular epidemiology

## Abstract

For several years after its discovery, Porcine circovirus 2 (PCV-2) represented a major threat to the swine industry through economic losses due to the associated clinical syndromes, decreased production performances in both symptomatic and asymptomatic animals and disease management costs. Widespread vaccination administration has largely reduced the impact of this infection and represents the most effective control measure. The efficacy of vaccination is threatened by the emergence of novel (or uncommon) PCV-2 genotypes. In addition to domestic pigs, PCV-2 has been detected in several other species, a fact which could have an impact on new variant emergence and maintenance. Considering this, the present study assessed the distribution of the minor PCV-2c genotype in non-Suidae ungulates in Namibia. Red hartebeests (*Alcelaphus buselaphus caama*) (n = 44), kudus (*Tragelaphus strepsiceros*) (n = 10) and oryxes (*Oryx gazella*) (n = 54), whose mediastinal lymph nodes were sampled after slaughtering during the period 2019–2021, were included in the study. Two oryxes (3.7%; 95% CI = 0.45–12.75%) were PCV-2-positive by PCR. Complete genome sequence was obtained for the two samples identifying them as PCV-2c genotype. The sequences were identical and shared a high percentage of identity (~99.9%) with those recently obtained from warthogs living in the same area. The present study confirms the presence of the PCV-2c genotype (previously considered extinct) in Namibian wild animal populations and demonstrates greater than expected PCV-2 host plasticity. Because of the role these niches can have in the maintenance and evolution of minor PCV-2 genotypes, more extensive and dedicated studies should be performed to prepare authorities to promptly react to potential emerging threats from these viruses.

## 1. Introduction

The genus *Circovirus*, family *Circoviridae,* is a viral group that has been known since the early 1970s when the first member of this group, *Porcine circovirus* 1 (PCV-1), was identified [1]. Circoviruses are small, non-enveloped viruses with a single-stranded circular DNA genome of approximately 2 kb encoding at least two proteins, the capsid (Cap) and a replicase (Rep). Additional proteins have been identified for some species and are involved in the host cellular cycle, signaling and immune response regulation [2,3]. 

Following the identification of PCV-1, a number of circovirus species were identified in different hosts, and currently, 49 species are classified in the genus *Circovirus* (https://talk.ictvonline.org/taxonomy/ accessed on 1 November 2021). In recent years, most of these identifications have been obtained using degenerate, universal primers or metagenomic approaches. For this reason, the virus–host association has only been reliably established for a limited number of species. Caution should be applied, especially with viral sequences obtained from samples associated with the digestive tract or from hematophagous parasites, since the source of detected viral DNA could be an infected meal [3].

In most instances, no association has been established between circovirus infection and overt clinical signs. However, some circovirus infections have been shown to be responsible for clinically relevant syndromes among birds, carnivores and especially pigs [4,5,6].

It was with the emergence of Porcine circovirus type 2 (PCV-2) in the 1990s that circoviruses, initially largely neglected, attracted more attention because of the huge economic impact caused by associated clinical syndromes and decreased production performances in both symptomatic and asymptomatic animals [7]. In pigs, PCV-2 infection has been associated with severe clinical syndromes, broadly referred to as porcine circovirus diseases (PCVD), which include PCV2 systemic disease (PCV2-SD), PCV2 reproductive disease (PCV-2-RD), Porcine dermatitis and nephropathy syndrome (PDNS) [8]. PCV-2 is characterized by a remarkable genetic variability originating from both high mutation and recombination rates that, in combination with natural selection and particularly immune-induced selective pressures, have shaped PCV-2 strain phenotype and genotype, leading to the emergence of different variants, currently classified into nine genotypes (from PCV-2a to PCV-2i) [9,10,11]. However, just three of these genotypes have demonstrated a worldwide distribution [10]. The first genotype to be detected, and initially the most prevalent, was PCV-2a, which was progressively replaced (referred to as the “first genotype shift”) [12,13] by PCV-2b around the beginning of the new millennium. PCV-2b was, in turn, largely superseded by PCV-2d since 2008–2010 (second genotype shift) [9,14]. The other genotypes have only been sporadically reported in certain geographical areas; the reasons behind the different fitness and epidemiological success of some genotypes over others are still not understood. The prevalence of these “minor” genotypes and how they are maintained are similarly obscure. 

The history of one of these, PCV-2c, is especially exemplifying. PCV-2c was initially reported in 2008 from Danish archival samples collected between 1980 and 1990 [12]. The genotype was considered extinct until 2015, when Franzo et al. demonstrated its presence in a feral pig population in the Pantanal region of Brazil [15] and evidence of its circulation in domestic pig in China was also provided [16]. More recently, PCV-2c strains, although distantly related to the Brazilian and Danish strains, were detected in warthogs from two contiguous livestock farms in Windhoek, Kharas region, Namibia [17]. 

These findings stress the role of neglected ecological and geographical niches in maintaining minor and/or potentially novel circovirus genotypes over long time periods, a phenomenon that could represent a threat to domestic pigs and the possibility of the emergence of new or the re-emergence of previously known genotypes.

Despite the fact that circoviruses have been traditionally considered largely host-specific, growing evidence seems to contradict this view, supporting a higher than expected host plasticity [18,19]. PCV-2c detection in warthogs is not an exception since PCV-2 has also been detected wild boar, cattle, goats, mink, raccoon dogs and foxes, although the clinical relevance in these species is a matter of discussion (Zhai et al., 2019). Similarly, another member of porcine circoviruses, Porcine circovirus 3, has been occasionally detected in ruminants, rodents, canines and insects [18]. Similar conclusions can be drawn for other circoviruses such as Beak and Feather Disease virus [20,21] or canine circovirus [22].

To assess the distribution of the neglected PCV-2c genotype (and potentially other PCV-2 genotypes) in unexpected host species, a study was performed on wild, *non-Suidae* ungulates in Namibia sharing the same environment, risk factors and infectious pressure of the warthog populations previously demonstrated to harbor PCV-2c (Molini et al., 2021). 

## 2. Results

**Figure 1:**
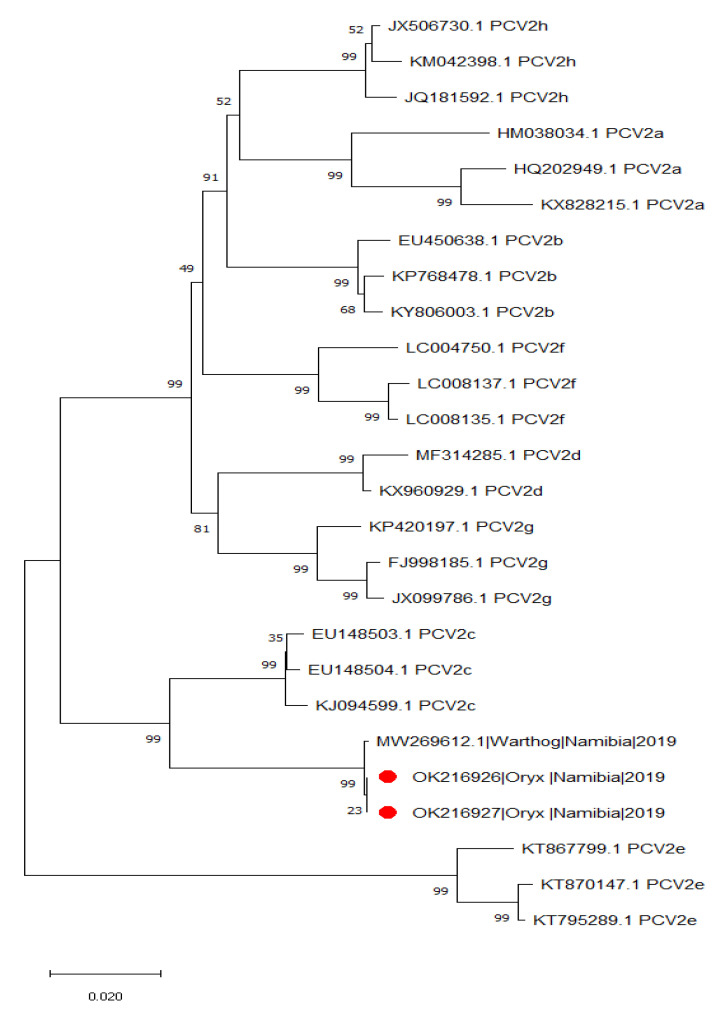
Neighbor-joining phylogenetic tree reconstructed using the complete ORF2 gene sequences of PCV-2 strains herein described (full red circles), plus a subset of the reference sequences available from Franzo and Segalés [10].

All red hartebeest (*Alcelaphus buselaphus caama*) (0/44; 0%) and kudu (*Tragelaphus strepsiceros*) (0/10; 0%) were PCV-2-negative. However, two out of 54 (3.7%; 95% CI = 0.45–12.75%) oryx (*Oryx gazella*) were PCV-2-positive (Table 1) at a low to moderate viral titer (i.e., 1.56 × 10^3^ and 5.45 × 10^4^ copies/mL). The complete genome sequence was obtained for both samples. After comparison with PCV-2 reference strains, they were classified within the PCV-2c genotype. The oryx PCV-2c sequences were identical and shared a high percentage of identity (~99.9%) with those obtained from warthogs living in the same area (Molini et al., 2021). Phylogenetic analysis using the full ORF2 dataset described in Material and Methods confirmed the high similarity of the oryx sequence with those of the warthogs (Figure 1). The genetic distance compared to Danish and Brazilian PCV-2c strains was between 4.4% and 4.6% at the genome level, 5.9% and 6.1% in the ORF2 and 3.17% and 3.81% in the ORF1, respectively. A two-nucleotide deletion, shared by warthogs and oryx strains, was present in the intergenic region between ORF1 and ORF2. 

**Figure 2:**
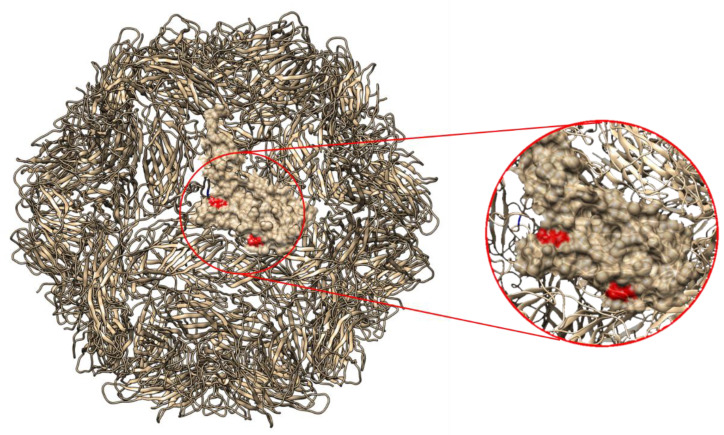
PCV-2 capsid ribbon structure reconstructed using homology modeling. The surface of one Cap protein was estimated and the amino acids differentiating Danish/Brazilian from the Namibian strains are highlighted in red.

At the amino acid level, three mutations differentiating Danish/Brazilian strains from the Namibian strains were present at positions 136 (L → Q), 166 (V → I) and 183 (L → I). Amino acids 136 and 166 were shown by homology modeling to be exposed on the viral surface (Figure 2). In the Rep, a four-amino acid insertion was identified (i.e., EEGM) starting at position 47 of PCV-2c reference strain (EU148503). Two mutations in the Rep differentiated Namibian stains from the Danish/Brazilian strains at position 139 (H → I) and 294 (E → A), (using EU148503 as a reference). The other mutations present in the Namibian PCV-2c compared to the reference strain were not unique as they were also present in either the Brazilian or Danish strains. 

## 3. Discussion

Since its first identification, PCV-2 has become one of the most relevant infectious diseases of the swine industry. The development of effective vaccines remarkably reduced the clinical impact of PCVD and the productive losses associated with asymptomatic infections, as well [7,23]. For this reason, PCV-2 vaccination is one of the biggest achievements of the pig industry in recent decades and PCV-2 vaccines are currently the most sold preventive products for swine worldwide [7]. Currently available commercial vaccines are, with few exceptions, based on the capsid of PCV-2a [24]. The most concerning issue regarding PCV-2 vaccination is thus the risk of new emerging variants that might partially or totally escape vaccine-induced immunity, as previously reported [7,23,25,26,27]. Moreover, no studies are available on the effectiveness of vaccination against minor genotypes. Based on these considerations, ecological niches that can host neglected/minor/unknown genotypes must be considered as a relevant threat. Widespread PCV-2 circulation has been recently demonstrated in Africa including minor genotypes such as PCV2-c and PCV-2g (Franzo et al., personal communication; Molini et al., 2021). Considering the increasing trade and intercontinental connections of many African countries, these reports deserve particular attention. 

The present study confirms the presence of PCV-2c in Africa and identified it in oryx. When confronted with such unexpected results, potential contamination and false-positive results must be confidently excluded. For this reason, samples were collected respecting a protocol designed to minimize contamination risks. Additionally, sample positivity was confirmed using a different assay in the University of Padova laboratories, where PCV-2c has never been introduced nor detected. Finally, although similar, the sequences obtained from the oryx were different from those reported in warthogs. Based on these criteria and findings, we believe that the risk of cross-contamination can be reasonably excluded

PCV-2c identification in wild or feral species only could suggest that this genotype somehow benefits from a lower competition from other genotypes that are circulating in domestic, intensively raised pigs. Alternatively, host or population adaptation of PCV-2c could be speculated. Although the prevalence of PCV-2c was lower than that observed in warthogs [17], this genotype’s circulation in oryxes is noteworthy and testifies the remarkable host plasticity of PCV-2. The high genetic identity between the PCV-2c strains collected from warthogs and oryxes, coupled with the shared environment, supports transmission between the two species rather than the existence of isolated, host-specific viral populations. PCV-2 infection has already been reported in domestic bovines [28,29]. A relatively low host barrier among artiodactyl to PCV-2 infection could therefore be speculated, and together with close proximity due to shared environments, an artificially high infectious pressure favoring a host jump could have been imposed.

PCV-2 attachment to the cell surface is mediated by glycosaminoglycans (GAGs) heparan sulfate (HS) and chondroitin sulfate B (CSB) [30], which might be less variable between hosts than other cell receptors. Additionally, previous research has revealed that PCV-2 could still infect mutant hamster ovary (CHO) cells that did not express GAGs, indicating that other yet unidentified cellular surface molecules contribute to PCV-2 binding to the host cell [31]. Therefore, despite not being fully understood, the PCV-2 infection mechanism could favor entrance into different host cells [30]. The clinical relevance of PCV-2 infection in *Bovidae* remains elusive. In the present study, positive animals were in good health and viral titers were low, compatible with subclinical infection. Nevertheless, effect on animal growth, susceptibility to other infections and welfare cannot be excluded. Additionally, the limited sample size and infection level did not allow for the study of the potential impact of other co-factors in clinical disease emergence in these species. Why the other *Bovidae* species (i.e., red hartebeest and kudu) were not infected is not known. Other host barriers or differences in interactions between warthogs and the *Bovidae* species might affect individual exposure and infection risk. Nevertheless, the small sample size tested in this study does not allow for clear conclusions on PCV-2c infection of the species investigated. Future studies will require more epidemiologically robust sampling approaches.

The Namibian PCV-2c strains were significantly different compared to the previously identified strains from Denmark and Brazil. Whether such genetic and phenotypic difference is due to geographical isolation and independent evolution or host adaptation is currently impossible to say. Of note, three amino acid mutations differentiating the two groups (Namibia vs. Denmark/Brazil) were identified in the Cap, two of which (residues 136 and 183) are exposed on the viral surface and are located in potentially immunogenic regions [32,33,34]. Moreover, Dhindwal et al. demonstrated that the sulfates of heparin oligosaccharides are close to amino acids 58, 59, 63, 73, 89, 128, 132, 186, 188 and 227 of the Cap. Experimentally, single substitutions of these amino acids to alanine diminished the capsid’s affinity for heparin [30]. Variation in amino acid 136 and 183 could, therefore, have a similar impact on the capsid’s affinity for heparin because of their proximity to the above-mentioned amino acids [35]. 

## 4. Materials and Methods

### 4.1. Sample Collection and PCV-2 Diagnosis

The study included mediastinal lymph nodes from 108 slaughtered wild ruminants (Table 1) collected between 2019 and 2021 from the abattoir of a livestock farm in Windhoek, Kharas region, Namibia. The ruminants were hunted in the morning (from 5 AM to 11 AM) and slaughtered inside the university farm. They were then immediately transported to the farm abattoir where they were processed and the carcasses were stored at 4 °C. All the operations were performed before the end of the day according to standard operation procedures for hygiene of the University of Namibia. (Note: Although the same abattoir was used for the slaughter of warthogs previously shown to be positive for PCV-2c (Molini et al., 2021), due to public health requirements, the wild ruminants investigated in this study were slaughtered in a different area of the abattoir and on separate days from the warthogs.) 

The mediastinal lymph nodes were collected during the post mortem inspection using a disposable scalpel and a sterile container for each individual carcass. The tissue was homogenized in 1 mL of sterile phosphate-buffered saline (PBS) using a TissueLyser LT (QIAGEN, Hilden, Germany). Total genomic DNA was extracted using a High Pure Viral Nucleic Acid Kit (Roche, Basilea, Switzerland) with an elution volume of 100 μL, following the manufacturer’s instructions. PCV-2 presence was tested by PCR using the primers P1 (forward) (5′-TAA TCC TTC CGA AGA CGA GC-3′) and P2 (reverse) (3′-CGA TCA CAC AGT CTC AGT AG-5′) to amplify a fragment of 629 base pairs (bp) of ORF1 (the replication-associated protein gene) [36]. Positive results were confirmed and viral titers were determined at the Laboratory of Veterinary Infectious Diseases, Department of Animal Medicine, Production and Health, University of Padova, where they were tested by quantitative real-time PCR [37].

### 4.2. Complete Genome Sequencing and Analysis

The complete genome of positive samples was amplified using three PCRs based on the additional pairs of primers: P3 (forward) (5′-CAG AAG CGT GAT TGG AAG AC-3′) and P4 (reverse) (3′-ATG TAG ACC ACG TAG GCC TC-5′) (pair 2); P5 (forward) (5′-AGA AGC TCT TTA TCG GAG GA-3′) and P6 (reverse) (3′-AAG CGA ACC ACA GTC AGA AC-5′) (pair 3); and P7 (forward) (5′-CTA GAA TAA CAG CAC TGG AG-3′) and P8 (reverse) (3′-GTT CGT CCT TCC TCA TTA CC-5′) (pair 4). Four overlapping fragments covering the entire PCV-2 genome were obtained. Briefly, the PCR reaction conditions consisted of 5 μL of extracted DNA in a final reaction volume of 20 μL containing a final concentration of 1.25 mM MgCl_2_, 1X PCR buffer, 0.2 mM dNTPs, 10 pmol of each primer and 2.5 U of Taq DNA polymerase. All the reactions were performed with the following thermal profile: initial denaturation at 94 °C for 5 min and then 35 cycles of denaturation at 95 °C for 30 s, annealing at 51 °C for 30 s and elongation at 72 °C for 60 s, followed by a final elongation at 72 °C for 5 min [36]. PCR amplicons were purified using a Wizard SV Gel and PCR Clean-Up System (Promega) and sequencing was performed at LGC Genomics (Berlin, Germany). The sequences (submitted to the GenBank database under accession numbers OK216926 and OK216927) were edited and assembled using the Staden software package version 2.0.0b8 and aligned to the full ORF2 reference dataset described by Franzo and Segalés (2018) at the codon level using the MAFFT method implemented in TranslatorX [38]. A neighbor-joining phylogenetic tree was reconstructed based on the raw genetic distances (pairwise p-distance) using MEGA7 to genotype them [39]. The robustness of inferred clades was assessed by performing 1000 bootstrap replicates. 

### 4.3. Capsid Protein Homology Modeling

The translated protein sequence of one PCV-2c strain collected from oryx was submitted to the SWISS-MODEL webserver to select the best template for which experimentally determined quaternary structure was available [40]. The same program was used to determine the protein structure through a homology modeling approach. The obtained model was visualized and edited with Chimera [41].

## 5. Conclusions

In conclusion, the present study confirms the presence of the PCV-2c genotype (previously considered extinct) in Namibian wild animal populations, although in the context of a semi-intensive farming system. The infection of *Bovidae* species sharing the same environment of an infected *Suidae* population testifies a greater than expected PCV-2 host plasticity, although the actual consequences from a clinical and/or animal welfare perspective remain unknown. Nevertheless, because of the relevance these niches can have in the maintenance and evolution of minor PCV-2 genotypes, against which the protection induced by currently available vaccines has not been properly investigated, further extensive and dedicated studies should be performed to fill such knowledge gaps and prepare authorities to promptly react to potential threats to the swine industry from emerging porcine circoviruses. 

## Figures and Tables

**Table 1:** Summary of tested and positive samples collected from different hosts between 2019 and 2021.

Year	Species	Number of Samples Tested	Positive Samples
2019	Red hartebeest (*Alcelaphus buselaphus caama*)	34	0
	Kudu (*Tragelaphus strepsiceros*)	2	0
	Oryx (*Oryx gazella*)	36	2
2020	Red hartebeest (*Alcelaphus buselaphus caama*)	3	0
	Kudu (*Tragelaphus strepsiceros*)	2	0
	Oryx (*Oryx gazella*)	2	0
2021	Red hartebeest (*Alcelaphus buselaphus caama*)	7	0
	Kudu (*Tragelaphus strepsiceros*)	6	0
	Oryx (*Oryx gazella*)	16	0

## Data Availability

The data that support the findings of this study are openly available in GenBank, reference numbers OK216926 and OK216927.
